# Like a Bolt from the Blue: Phthalocyanines in Biomedical Optics

**DOI:** 10.3390/molecules17010098

**Published:** 2011-12-23

**Authors:** Nawal Sekkat, Hubert van den Bergh, Tebello Nyokong, Norbert Lange

**Affiliations:** 1 School of Pharmaceutical Sciences, University of Lausanne/Geneva, Geneva, 30, quai Ernest Ansermet, Geneva CH-1211, Switzerland; 2 Laboratory of Photomedicine, Swiss Federal Institute of Technology (EPFL), Lausanne CH-1015, Switzerland; 3 Department of Chemistry, Rhodes University, Grahamstown 6140, South Africa

**Keywords:** biomedical optics, fluorescence diagnosis, phthalocyanines, NIR dyes, photodynamic therapy

## Abstract

The purpose of this review is to compile preclinical and clinical results on phthalocyanines (Pcs) as photosensitizers (PS) for Photodynamic Therapy (PDT) and contrast agents for fluorescence imaging. Indeed, Pcs are excellent candidates in these fields due to their strong absorbance in the NIR region and high chemical and photo-stability. In particular, this is mostly relevant for their *in vivo* activation in deeper tissular regions. However, most Pcs present two major limitations, *i.e.*, a strong tendency to aggregate and a low water-solubility. In order to overcome these issues, both chemical tuning and pharmaceutical formulation combined with tumor targeting strategies were applied. These aspects will be developed in this review for the most extensively studied Pcs during the last 25 years, *i.e*., aluminium-, zinc- and silicon-based Pcs.

## 1. Introduction

The first syntheses of metal-free and copper phthalocyanines were reported in 1907 by Braun and Tcherniac at the South Metropolitan Gas Company (United Kingdom) and in 1927 by Diesbach and von der Weid at the University of Fribourg (Switzerland). A few years later, Professor Linstead in collaboration with Imperial Chemical Industries (ICI) was the first to characterize the chemical structure of the phthalocyanine molecule, using for the first time the term “phthalocyanine” [1,2,3].

Nowadays, phthalocyanines are widely used in the dying industry. Nearly a quarter of all pigments of organic origin are related to this class of compounds. Furthermore, they are used for the fabrication of high-speed and high resolution optical media [4], as light harvesters in photovoltaic applications [5], and as experimental catalysts in redox reactions [6]. These dyes absorb strongly in the red and near infrared (NIR) part of the visible spectrum providing them with their characteristic blue or greenish color. Pcs that absorb in the NIR are especially interesting for photomedical applications such as fluorescence imaging, Photochemical Internalisation (PCI), and Photodynamic Therapy (PDT) [7,8,9,10,11,12].

Just recently Photochemical Internalisation (PCI), a novel drug delivery process, has shed light on the importance of phthalocyanines and their applications in oncology [13,14,15]. The PCI technology is based on the concomitant administration of a therapeutic agent and a photosensitizer. When internalized by endocytosis and consequently colocalized in the endosomes and/or lysosomes, light activation of the PS will subsequently lead to vesicle disruption and release of the therapeutic agent. Indeed, PCI technology enables the release of endocytosed drugs prior to lysosomal degradation, thus, increasing their therapeutic efficacy within the target cells. Among the photosensitizers used in PCI, the amphiphilic disulfonated aluminium phthalocyanine AlPcS_2adj_ displays all the required features and characteristics such as specific insertion of the hydrophobic part of the PS into the endocytotic membrane. Due to its amphiphilic character, AlPcS_2adj_ “intrudes into the plasma cell membrane, but is unable to penetrate through the plasma membrane and will enter the cells via adsorptive endocytosis” [16]. The concomitant administration of a drug such as gelonin or bleomycin [14,17,18] will, after internalization lead to vesicle disruption and intracellular release of the drug upon light activation [14,19,20].

As mentioned previously, phthalocyanines strongly absorb in the NIR, and have been proposed for PDT of cancer as early as 1985 [21,22]. Under some circumstances PDT treatment presents several advantages over conventional cancer therapies such as chemo, radiation and surgical treatments. It enables selective destruction of malignant tissues due to specific interaction of three individually non-toxic components *i.e*., a photosensitizer (PS), light and oxygen. PS selective accumulation in tumor combined with its controlled light activation enables selective destruction of tumors, sparing neighboring healthy tissue.

Although today the selectivity of PS is not fully understood, it is thought to be a multifactorial process including physico-chemical properties and binding to plasma proteins as well as the particular characteristics of tumors such as leaky vasculature, low lymphatic drainage, expression of specific enzymes and receptors and pH variation. Depending on its cellular and intracellular localization/relocalization, PS exhibits direct and indirect cell killing, vascular occlusion, release of cytokines and the response of the immune system [23,24].

Photofrin^®^ (Table 1) was the first photosensitizer approved for clinical use in 1993 for the treatment of bladder cancer. Since then it gained marketing for the prophylactic treatment of several cancers such as the treatment of early-stage oesophagal, gastric, cervical and lung cancers [24,25]. However, this first generation photosensitizer has several limitations with respect to its clinical use since it [23,25,26,27,28]:

• Is composed of a undefined mixture of hematoporphyrin derivatives (HpD);
• Induces a long-lasting skin photosensitization (2 to 3 months post injection);
• Has a low extinction coefficient at wavelengths for optimal tissue penetration;
• Displays a limited selectivity for the target tissue.

Therefore, considerable efforts have been undertaken to prepare 2nd generation PS with more suitable features [29,30,31] such as:

• Single and chemically pure compound;
• Stability and good solubility in pharmaceutically acceptable formulations and in biological media;
• Low tendency to aggregate;
• High singlet oxygen quantum yield;
• Photostability;
• Fluorescence;
• Low phototoxicity towards healthy tissue;
• No dark toxicity;
• Fast clearance from the healthy parts of the body and specific retention in diseased tissues;
• Strong absorbance in NIR region and minimal absorbance between 400 and 600 nm.

In order to avoid skin photosensitization, the PS has to present the lowest absorbance in the spectral range where daylight intensity is the highest, *i.e*., between 400 and 600 nm. Moreover, strong absorbance in the NIR region between 600 and 900 nm favors the optimal penetration of light into tissues, thus resulting in more efficient PDT effects when treating deeper lying lesions. However, absorption by water molecules increases for wavelengths above 800 nm and at higher wavelength energy transfer to molecular oxygen is suboptimal. Hence, the window for optimal light penetration ranges from 600 to 800 nm. These considerations are based on the assumption that healthy and diseased tissues have the same absorbance of light, which in reality is not the case. Finally, 94 kJ/mol appears to be the minimal energy required by a photon to induce singlet oxygen ^1^O_2_ formation. This energy corresponds to a wavelength of approximately 1,270 nm [32,33,34,35,36,37,38,39].

**Figure 1:**
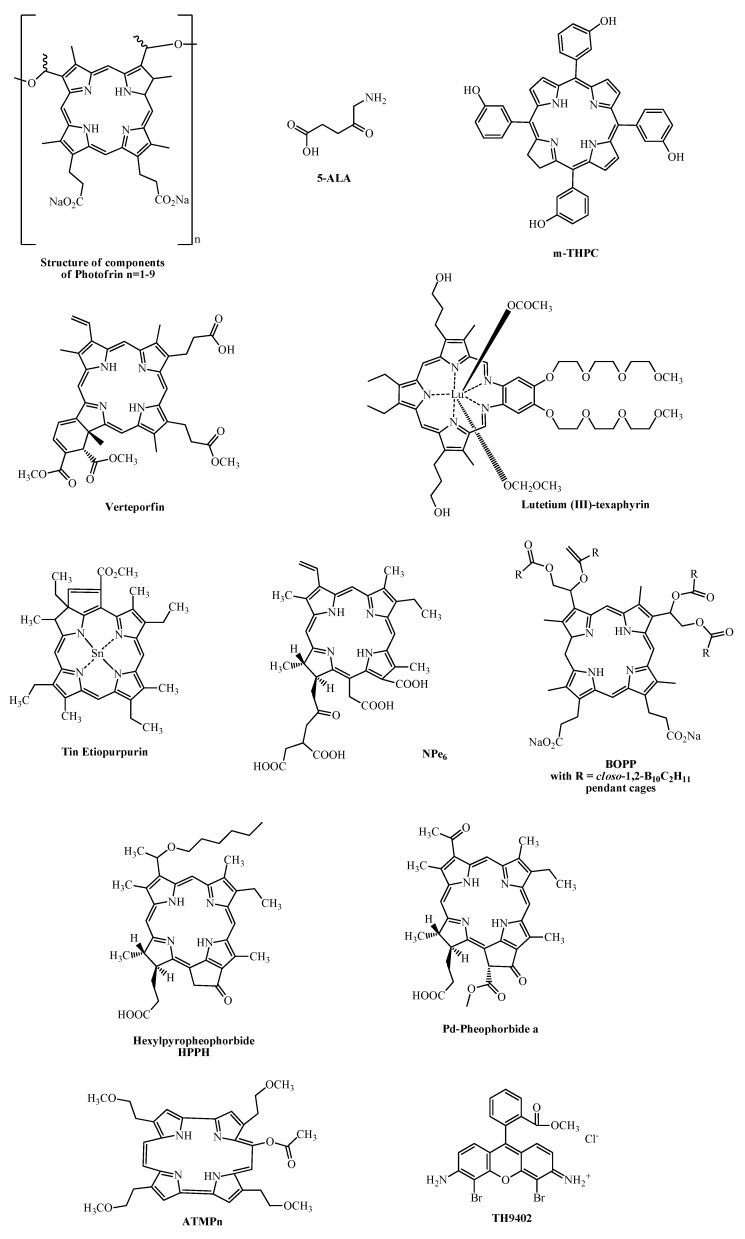
Chemical structures of clinically relevant “non-phthalocyanine” photosensitizers.

In Table 1 some 1st and 2nd generation PS investigated in clinical trials and their potential therapeutic application in oncology are listed (see also Figure 1 for their chemical structures).

**Table 1:** Examples for PS used in clinical and preclinical trials in oncology [24,25,40,41,42,43,44,45,46,47,48,49,50,51,52].

Photosensitizers	Trade Name	Absorption Wavelength	Potential Indications
HpD, Porfimer sodium	Photofrin, Photogem, Photosan, Hemporfin	630 nm	Cervical, brain, oesophageal, breast, head and neck, lung, bladder, superficial gastric cancers, Bowen's disease, cutaneous Kaposi's sarcoma
m-THPC, Temoporfin	Foscan	652 nm	Oesophageal, prostate and pancreatic cancer, advanced head and neck tumors
Verteporfin	Visudyne	689 nm	Basal and squamous cell carcinomas
HPPH, 2-(1-hexyloxyethyl)-2-devinyl pyropheophorbide-alpha	Photochlor	665 nm	Basal cell carcinoma, Oesophageal cancers, Head and Neck tumors
Palladium-bacteria-pheophorbide	Tookad	763 nm	Prostate cancer
5-ALA,5-aminolevulinic acid	Levulan	630 nm	Skin tumors, head and neck, gynaecological tumors and basal cell carcinomas
		375-400 nm	Brain, head and neck and bladder cancer photodetection
5-ALA-methylester	Metvix	635 nm	Basal cell carcinoma
5-ALA benzylester	Benzvix	635 nm	Gastrointestinal tumors
5-ALA hexylester	Hexvix	375-400 nm	Photodectection of bladder cancer
Lutetium (III)-texaphyrin or Motexafin-lutetium	Lutex, Lutrin, Antrin, Optrin	732 nm	Prostate, cervical, breast, brain cancer, melanoma
SnET2, Tin (IV) ethyl etiopurpurin	Purlytin, Photrex	659 nm	Kaposi's sarcoma, cutaneous metastatic adenocarcinomas, prostate, brain, lung cancers, basal cell carcinomas
NPe6, mono-L-aspartyl chlorin e6, talaporfin sodium	Talaporfin, Laserphyrin	664 nm	Solid tumors, lung cancer, cutaneous malignancies
BOPP, boronated protoporphyrin	BOPP	630 nm	Malignant gliomas
Zinc phthalocyanine	CGP55847	670 nm	Squamous cell carcinoma of upper aerodigestive tract
Silicon phthalocyanine	Pc 4	675 nm	Cutaneous and subcutaneous lesions from diverse solid tumor origins
Mixture of sulfonated aluminium phthalocyanine derivatives	Photosens	675 nm	Skin, breast, lung, oropharingeal, breast, larynx, head and neck cancers, Sarcoma M1, epibulbal and choroidal tumors, eyes and eyelids tumors, cervical cancer
ATMPn, Acetoxy-tetrakis(β-methoxyethyl)-porphycene	NA	600-750 nm	Skin cancer
TH9402, dibromorhodamine methyl ester	NA	515 nm	Breast, myeloma, non-melanoma skin cancer

Another appealing feature of Pcs is the ease of the introduction of peripherial and axial substituents that can be used to fine tune absorption and emissions characteristics. Phthalocyanine dyes with absorptions bands as high as 1,000 nm with suitable fluorescence quantum yields can be prepared [11]. Therefore, Pcs are potentially interesting as fluorescence reporters for *in vitro* and *in vivo* imaging that can replace commonly used fluorophors such as fluorescein and indocyanine green. Two of such compounds, “La Jolla Blue^®^” and *“*IRD700DX^®^*”* are commercially available as labeling agents for proteins, peptides [53,54,55], antibodies [56,57,58,59,60] and oligonucleotides [61,62,63,64,65].

This review intends to compile preclinical and clinical data accrued with Pcs as PS for PDT. However, it has to be noted that the presented concepts can be easily translated into the use of similar compounds for the fluorescence diagnosis of disease. Thereby, we will mainly focus on the most extensively studied Pcs, *i.e*., Pcs containing Al, Zn, and Si as central metal ion. At first we will briefly describe how chemical modulation of Pcs, alters their Structure-Activity Relationship (SAR). Then, since most Pcs are barely soluble in pharmaceutically acceptable formulations special emphasis will be placed on the impact of pharmaceutical formulation on their therapeutic efficiency. And finally, different tumor targeting strategies that have been exploited with Pcs will be discussed.

## 2. Phthalocyanines

**Figure 2:**
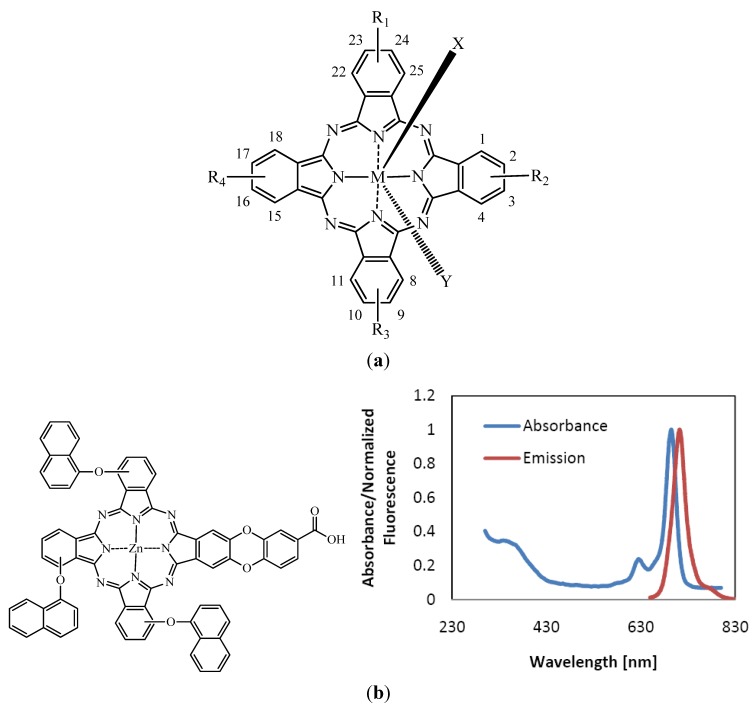
(**a**) General chemical structure of metallated phthalocyanines; (**b**) Typical absorption and emission spectra of metallated Pc (e.g., zinc based phthalocyanine in DMSO).

Pcs belong to the group of 2nd generation PS which exhibit a high extinction coefficient around 670 and 750 nm and even up to 1,000 nm. Variation of the axial and peripheral substituents modulates the tendency for aggregation, pharmacokinetics, biodistribution, solubility, as well as fine-tuning of NIR absorbance [66]. Extinction coefficients higher than 10^5^ M^−1^cm^−1^ have been reported [11,66]. These compounds are porphyrin-like PS, displaying tetrapyrrolic, aromatic macrocycles with each cycle linked to the other by nitrogen atoms. Each pyrrolic ring is extended by a benzene ring resulting in the red-shift of their final absorption band [31,67,68]. Figure 2 shows the general chemical structure of Pcs and nomenclature as well as typical absorption and emission spectra.

Lowering of the symmetry of the phthalocyanine molecules results in splitting (or broadening) of the Q bands. This splitting is due to the lifting of degeneracy of the lowest unoccupied molecular orbital (LUMO) to a varying extent. It is also well established that expansion of π conjugation in phthalocyanines shifts the Q band to the red. Extension of the conjugation system is accompanied by change in colour from blue/green to colors including brown, red or purple. The location of the Q band in Pc complexes can be adjusted by attaching suitable substituents onto the peripheral and non-peripheral positions of the ring and by the change in the nature, size and number of substituents. Addition of electron donating groups such as –NH_2_, OR and SR at the non-peripheral (1,4,8,11,15,18,22,25) or peripheral (2,3,9,10,16,17,23,24) positions of the Pc ring results in red shift to the NIR region. Substitution at the non-peripheral position shows more red-shift than at the peripheral position [69].

Besides their strong absorption in the NIR, Pcs exhibit low absorption at wavelengths between 400 and 600 nm leading potentially to a lower skin photosensitization when exposed to sunlight [31]. Moreover, the presence of a diamagnetic central metal such as Zn^2+^ and Al^3+^ in the Pc nucleus seems to improve the triplet state life time (τ_t_), as well as its yield (Ф_t_) and singlet oxygen yields (Ф_Δ_) compared to paramagnetic metals (e.g., Ф_Δ_ ≥ 0.7, Ф_t_ > 0.4 and τ_t_ = 750 μs for AlPcS_4_ in solution with human serum albumin) [30,48,67,70]. However, the metallation is not required for its photodynamic activity. Indeed, Feofanov *et al*. [71] and Karmakova *et al*. [72] reported efficient antitumor activity of a mixture of metal-free sulfonated phthalocyanines with an average number of sulfonated groups of 2.4 *in vitro* on human epidermoid carcinoma cells HEp2 and *in vivo* on mice bearing murine P-388 lymphoma cells, respectively.

On the other hand, Pcs with good fluorescence quantum yields up to 0.6 have been reported. This, together with their high photostability compared to commonly used labeling agents should have placed this group of compounds into the upper part of the list of NIR fluorescence reporters. As an example IR700DX^®^ is about 20 times more stable than Cy5.5 at similar irradiation intensities and wavelengths. However, despite these photophysical and spectral characteristics the use of Pcs as labeling agents for fluorescence imaging or in PDT is still limited.

### 2.1. Aluminium Based Phthalocyanines

#### 2.1.1. Aluminium Sulfonated Phthalocyanines—SAR

Sulfonated phthalocyanines bearing a central aluminium ion have been extensively studied *in vitro* as well as *in vivo*. They display potent photodynamic activity on cell lines including G361 human melanoma cells [73], pancreatic carcinoma cells (H2T) [74], and human fibrosacroma cells (HT-1080) [75], as well as *in vivo* on rat (CBH rats) bearing fibrosarcoma (HSN/TC/7) [76]. These early studies indicated a strong dependence of the photodynamic efficiency on the degree of sulfonation, later systematically assessed by Chan *et al*. [77,78,79].

At this point one has to keep in mind that earlier reports on aluminium sulfonated phthalocyanines can be sometimes misleading due to the designation as AlPcS_4_ or as AlPcS of the commercially available product from Ciba-Geigy, which nevertheless consisted of a mixture of mono, di-, tri- and tetrasulfonated chloroaluminium phthalocyanines with an average degree of three sulfonated substituents. Thus, in this review, we will refer to this mixture as AlPcS, whereas AlPcS_4_ will be used to qualify the pure tetrasulfonated Pc, keeping in mind that even these more defined compounds will be mixtures of regio- and stereoisomers. Furthermore, some early works did not disclose the identity of the axial ligands which could be chloride (Cl) as well as hydroxy (OH) groups. Since most reported studies focus on the effect of sulfonation with respect to PDT, no further distinction according to their axial ligand will be made here. Finally, the IC_50_ values reported in this review cannot be compared from a study to another since this value depends among other things on the applied light doses.

Interestingly, AlPcS_4_ has shown promising *in vivo* results in the treatment of malignant gliomas [80,81]. Wistar rats inoculated intracranially with C6 glioma cells, responded within 5 days to intravenous injection of AlPcS_4_ in saline solution at a dose of 5 mg/kg and irradiation 6 h post administration at a dose of 100 J/cm^2^. However, upon illumination at a light dose of 200 J/cm^2^, AlPcS_4_-mediated PDT neurological symptoms (e.g., brain damage and oedema) appeared, while light alone had no effect. Higher light doses lead to death in mice bearing intracerebrally implanted VMDk murine glioma cells as reported by Sandeman *et al*., presumably concomitant with hyperthermic effects in the irradiated areas [82]. 

#### 2.1.2. Influence of the Degree of Sulfonation

In 1990, Chan *et al*. reported that *in vitro* monosulfonated chloroaluminium phthalocyanine (AlPcS_1_) was taken up faster, retained to a higher degree and showed higher phototoxicity in murine colorectal carcinoma (Colo-26) cells than higher sulfonated analogues (*i.e*., di, tri and tetrasulfonated chloroaluminum phthalocyanine; referred to as AlPcS_2_, AlPcS_3_ and AlPcS_4_ respectively) [77]. Furthermore, comparison of light doses necessary for efficient PDT on WiDr cells cultured in monolayers and as spheroids revealed that spheroids were less sensitive to PDT than cells grown in monolayers [78].

In contrast to *in vitro* experiments, an inverse tendency with respect to tumor accumulation and phototoxicity was observed when PDT was perfomed with this series of sulfonated Pcs *in vivo.* In BALB/c mice inocculated with Colo-26 tumors AlPcS_1_ had essentially no effect on tumor regression in contrast to the higher sulfonated compounds following the order AlPcS_4_ > AlPcS_3_ > AlPcS_2_ [77]. This discrepancy can be assigned to differences in pharmacokinetics and biodistribution of these compounds due to their differences in hydrophilicity [77,83]. As suggested by Chan *et al*., the fast clearance of highly sulfonated Pcs after intravenous administration can be circumvented by intraperitoneal application for these compounds [77].

Later, Chan *et al*. showed that AlPcS_2_ was more potent than AlPcS_3_ and AlPcS_4_, despite its lower accumulation in tumor xenografts. This suggests different PDT mechanisms of these compounds with respect to cell killing, vasculature occlusion, and intracellular localization [78]. Interestingly, the highest *in vitro* efficiency of AlPcS_2_ over other more hydrophilic aluminium sulfonated Pc was already demonstrated in 1988. The same tendency was later confirmed by Peng and Moan [83]. When injected intraperitoneally AlPcS_2_ was the most efficient PS followed by AlPcS_4_, Photofrin, and AlPcS_1_. The best efficiency was observed with a drug dose of 10 mg/kg and light exposure 2 h post-administration.

Moreover, the relative position of sulfonate group seems also to influence Pcs photoactivity. Indeed, AlPcS_2_ bearing adjacent sulfonated side-group (AlPcS_2adj_) rather than opposite side-substitution exhibited the best cell penetration and were the most phototoxic compounds amongst the sulfonated aluminium phthalocyanine serie, presumably due to their amphiphilic properties [84]. Therefore, the phototoxicity of mixed AlSPc *in vivo* should be related mostly to photoactivity of AlPcS_2adj_ isomers [78,83]. In a recently reported study, Mathews *et al*. compared the phototoxic effect of AlPcS_2adj_ to 5-aminolevulinic acid (5-ALA) on healthy brains in mice [85]. Based on PDT-induced and higher mortality rates, AlPcS_2adj_ was considered to be a more potent photosensitizer than 5-ALA. Moreover, in a comparative study, Gupta *et al*. [86] reported that liposomal formulation of AlPcS_2_ resulted in a higher phototoxic effect as compared to its free form on human glioma cells (BMG-1) despite its lower uptake by the cells. 

Despite the higher efficiency of AlPcS_2_ compared to other sulfonated aluminium phthalocyanines, extensive efforts and studies have been realized with the objective of further improving the pharmacokinetic properties and selectivity of the commercially available AlPcS_4_. 

**Figure 3:**
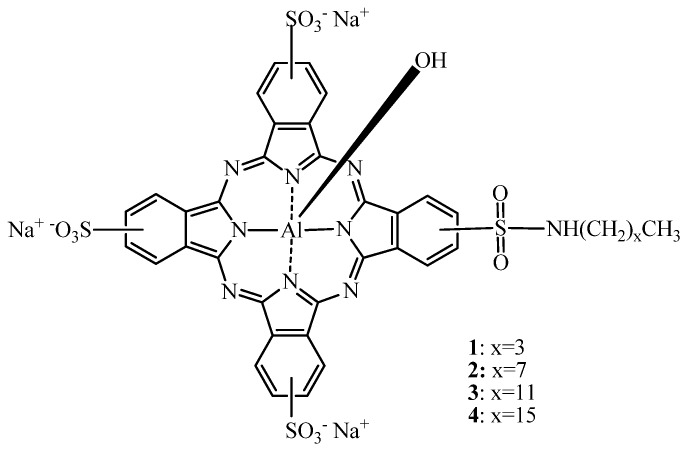
A homologous series of AlPc alkylsulfonamides.

Allen *et al*. [26], tested sulfonamides with an alkyl chain of 4, 8, 12 and 16 carbons to one of the sulfonated groups in AlS_4_Pc (Figure 3) and compared induced photodynamic effects to AlPcS_4_ in mice bearing EMT-6 tumors. They concluded that at doses of 0.2 μmol/kg, all tested AlPcS_4_-derivatives induced tumor regression and were more effective than AlPcS_2adj_ or AlPcS_4_. Moreover, with increasing alkyl chain length increased photodynamic efficacy was observed. In these experiments, AlPcS_4_ had no effect at doses up to 5 μmol/kg, and 1 μmol/kg of AlPcS_2adj_ was needed to induce 87% of tumor cure. 

#### 2.1.3. Photosens^®^

Photosens^®^, a mixture of aluminium chloride phthalocyanines with 1 to 4 sulfonated side-groups with an average sulfonated degree of 3, was developed at the General Physics Institute of the Russian Academy of Sciences. It has been evaluated *in vivo* for the treatment of sarcoma M1 [87], epibulbal and choroid tumors [88], eye and eyelid related tumors [89], bladder and cervical cancer [90,91]. In a clinical trial that included 47 patients composed of 35 women presenting pre-cancerous lesions of cervix and 12 women with non-invasive cervical lesions, a Photosens® dose of 0.3 mg/kg was applied. Twenty four hours post administration, lesions were irradiated with a light dose between 150 and 200 J/cm^2^ at 675 nm. No PDT-related pain was reported. Out of these two subgroups, 94.2% and 83.4% of the patients, respectively, responded fully to the treatment with complete tumor regression, with significant regression for 2.9 and 8.3% of the women. Moreover, the same percentage of women showed stabilized disease status. The remaining percentages correspond to the patients who responded only partially to the treatment. Today, Photosens^®^ is commercialized in Russia by NIOPIK.

#### 2.1.4. AlPcS-Conjugation to Tumor Targeting Moieties

Except for AlPcS_1_, aluminium sulfonated Pcs are readily soluble in aqueous media. Thus, most formulation efforts were aiming at increasing Pc accumulation and uptake by the tumors through the increase of the circulation half life or cellular/subcellular targeting.

The tetraglycine derivative Al(SO_2_N_gly_)_4_ and a mono-substitued AlPcS_4_ containing one 6-aminohexanoic acid spacer (AlPcS_4_A_1_) have been exploited for protein conjugation. The conjugation of AlS_4_Pc to monoclonal antibodies (Mab) lead to an enhanced phototoxicity in different cell lines such as human colon carcinoma LoVo [92], modified human ovarian carcinoma cells SKOV3-CEA-1B9 (SKOV cells transfected with carcinoembryonic antigen (CEA) cDNA and expressing two target antigens CEA and ErbB2) [60], and various squamous carcinoma cells including UM-SCC-11B, UM-SCC-22A, UM-SCC-22B, A431, SCV-7 and OE [56,93].

Moreover, *in vivo* biodistribution studies on tumor bearing mice revealed that these conjugates presented an increased selectivity toward the malignant tissues compared to free AlPcS_4_. Indeed, when 12 identical Pcs were conjugated to a 35A7 Mab directed against CEA (35A7 Mab-(AlPcS_4_A_1_)_12_), a tumor-to-muscle ratio and tumor-to-skin ratio of 33 and 8, respectively, was reported in nude mice bearing a human colon carcinoma xenograft [92]. In a subsequent study, the photodyanmic efficiency was further improved using an internalizing antibody (FSP77) directed against ErbB2. *In vitro*, SKOV3-CEA-1B9 showed a fast uptake of FSP77Mab-(AlPcS_4_A_1_)_6_ and a higher phototoxic efficacy (*i.e*., 96% *vs.* 68% of growth inhibition) as compared to the corresponding 35A7 Mab (AlPcS_4_A_1_)_8_ [60]. These studies are in agreement with the results reported by Vrouenraets *et al*. [56,94] Pcs conjugated to internalizing Mabs, *i.e*., SCC-Mab 425 and Mab U36, were photodynamically more effective than the corresponding non-internalizing Mab E48. Furthermore, the Mab 425-AlPcS_4_ was 7,500 times more effective *in vitro* than free AlPcS_4_. It has to be noted also that the loading of PS per Mab can be tuned through different conjugation methods. Indeed, by introducing a five carbon spacer chain between the Mab and AlPcS_4_, Carcenac *et al*. [92] were able to link up to 16 AlPcS_4_A_1_ entities to the proteins whereas Vrouenraets *et al*. could only conjugate four Al(SO_2_N_gly_)_4_ without affecting its solubility.

Later, Vrouenraets *et al*. tested three different Mabs [BIWA 4 (bivatuzumab), E48 and 425] on five different SCC cell lines (22A, 22B A431, SCV-7 and OE) [93]. PS conjugation to the aforementioned Mabs resulted in a higher phototoxicity compared to free AlPcS_4_. BIWA 4-AlPcS_4_ showed highest phototoxicity in all the five cell lines, exhibiting an IC_50_ as low as 0.06 nM as compared to IC_50_ ≥ 700nM for the free PS. Interestingly, the effect of the other Mabs varied depending on the cell line but not on the internalizing efficiency of the particular Mab. Mab E48-AlPcS_4_ conjugate was more active on 22A than on 431 cells, while Mab 425-AlPcS_4_ conjugate had the opposite effect. After determination of Pc’s cell binding and internalization, the authors suggested that the phototoxicity of these conjugates was more dependend on the overall cell binding than on their cellular internalization.

Overexpression of the low density lipoproteins (LDL) receptor has been reported for several tumors allowing the internalization of LDL proteins into the cell [95]. Therefore, Urizzi *et al*. [96] conjugated AlS_4_Pc to LDL either via covalent linking of a bisulfonated substituent of AlPcS_4_ through a 5-carbon spacer chain [(AlPcS_4_A_2_): aluminium di-(6-carboxypentylaminosulfonyl)-tetrasulfophthalocyanine) or non-covalent linking to the phospholipidic region of LDL (AlPcS_4_(C_12_)]. Loading ratios of 72:1 and 61:1 for AlPcS_4_A_2_-LDL and AlPcS_4_(C_12_)-LDL, respectively, have been reported to be the most stable formulations.

These compounds were tested *in vitro* on EMT-6 and A549 cells. After 2 h of incubation with the conjugates or with free AlPcS_4_, AlPcS_4_(C_12_)-LDL displayed a 10-fold higher phototoxicity effect than AlPcS_4_A_2_-LDL. This has been ascribed to the increased aggregation tendency, higher scavenging of ROS products, and alteration of the conjugate interaction with cellular receptors when Pc was covalently coupled to the protein.

Interestingly, free AlPcS_4_ and AlPcS_4_A_2_ were devoid of any photocytotoxic effects under the same conditions. A possible reason for this lack of photoxic activity could be the negative net charge of these Pcs, leading to a different cellular uptake and/or intracellular localization. 

Further *in vivo* studies on mice bearing EMT-6 allografts were performed comparing free AlPcS_4_ to AlPcS_4_(C_12_) and AlPcS_4_(C_12_)-LDL conjugate. No difference in tumor response was reported for both conjugates. As compared to free AlPcS_4_, 25 times lower drug doses could be used to induce similar responses. Moreover, the post-treatment oedema regressed within 3–4 days post-PDT. The similar phototoxic effect of free and AlPcS_4_(C_12_)-LDL conjugate can presumably be attributed to binding of the free Pc to plasma proteins (e.g., LDL) and redistribution post injection.

Overexpression of gastrin-releasing peptide-receptors (GRPR) have been associated with many cancerous conditions such as ovarian, breast, prostate and lung cancer [97]. Since bombesin is the amphibian homologue of the human gastrin releasing peptide [98] a recent study reported an attempt to target human prostate tumor cells PC-3 by conjugating AlPcS_4_ to bombesin. 

**Figure 4:**
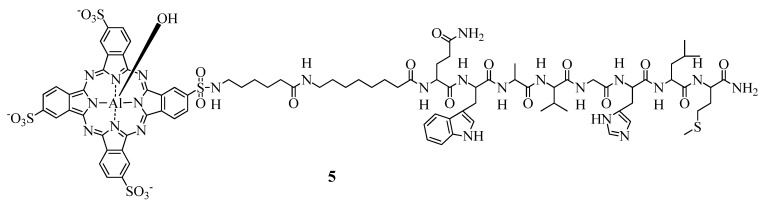
Chemical structure of the bombesin-AlPcS_4_ conjugate **5** for the targeting of GRPR.

However, due to the conjugation of bombesin to the AlPcS_4_ (Figure 4), the binding affinity to GRPR was about 40 times lower for the conjugate as compared to bombesin, certainly resulting in a loss of specificity and a moderate phototoxicity [99]. Nevertheless, the photoactivity of the conjugate was 2.5 and 5 fold higher than AlPcS_4_A_1_ and the free tetrasulfonated aluminium phthalocyanine, respectively, *in vitro*.

#### 2.1.5. AlPcS4-Formulation using Targeted Delivery Systems

Simple liposomes are usually unstable upon systemic administration and rapidly cleared from the body. However, the incorporation of PEG-derivatized phospholipids in the bilayer of liposomal membranes generally prolongs their circulation time. This property of PEGylated liposomes coupled to additional tumor targeting moiety such as transferrin was exploited by Gijsens *et al*. *in vitro* [100] and *in vivo* [101]*.* In HeLa cells, expressing approximatively 2 × 10^5^ transferrin receptors per cell [102], transferrin conjugated PEG-Liposomes containing AlPcS_4_ (Tf-Lip-AlPcS_4_) were 10 times more efficient than free AlPcS_4_ (IC_50_ of 0.63 μM *vs.* 6.3 μM, respectively) while non-Tf conjugated AlPcS_4_ liposomes (Lip-AlPcS_4_) were less potent than free PS. Indicating that non-targeted liposomes are only poorly taken up and additionally, show only limited release of their content into the cells [100].

Initially, the same tendencies were confirmed when these formulations were tested on AY-27 cells [101]. However, in a rat bladder model using the same cell line no or only moderate accumulation of all liposomal formulations was observed. However, pretreatment with a chondroitinase breaking the epithelial glycocalyx resulted in a selective retention of Tf-Lip-AlPcS_4_. On the contrary, free AlPcS_4_ was detectable with or without the enzymatic pretreatment of the bladder but with a lower selectivity. The reported tumor-to-normal tissue ratio and tumor-to-submucosa/muscle ratio for Tf-Lip-AlPcS_4_ were 18:1 and 78:1, respectively.

Qualls *et al*. [103] encapsulated AlPcS_4_ into pH sensitive liposomes using 1,2-di-O-(*Z*-1′-hexadecenyl)-*sn*-glycero-3-phosphocholine (DPPlsC) an acid labile lipid. Moreover, folate-polyethyleneglycol 3350-distearoylphosphatidylethalonamine (Folate-PEG3350-DSPE) was incorporated for targeting of folate-receptors in order to promote the receptor-mediated Pc internalization. The liposomes were subsequently tested *in vitro* on folate receptor expressing human nasopharyngeal cancer cells. It was found that DPPIsC-folate liposomes were taken up faster and were photodynamically more active than the free AlPcS_4_ confirming earlier studies with similar liposomes on colorectal carcinoma cells C170 cells by Morgan *et al*. [104]. Moreover, it was shown *in vitro* that the targeting moiety rather than the pH sensitivity of the delivery system dominated the photocytotoxicity of the PS [103].

#### 2.1.6. AlClPc—SAR

**Figure 5:**
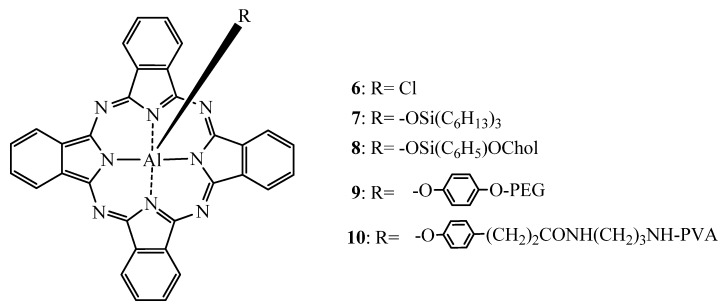
Chemical structure of AlPc **6–10** [106,107].

Modulation of the axial substituents is another way to modulate the pharmacological, pharmaceutical as well as optical properties of Pcs. As confimed by Chan *et al*., Ben Hur initially reported that a simple Pc carrying a chlorine as counterion (AlClPc, **6**, Figure 5) was photodynamically more active than its sulfonated counterparts [79,105]. In order to further improve these compounds systematic studies with different alterations of the axial substituents have been undertaken. Decreau *et al*. [106] reported increasing phototoxicity with increasing the hydrophobicity of AlClPc derivatives. Indeed, when incorporated into liposomal (Egg Yolk Lecithin based liposomes) or emulsion (Cremophor EL based emulsion) formulations, the hydrophobic aluminium Pc (AlPc) derivatives such as **7** and its cholesterol derivative **8** (Figure 5) were more phototoxic than **6**. After 1 h incubation of achromic M6 melanocytes with liposomal formulations of **6** or its derivatives the drug dose required to induce 50% of the cell death was 10^−8^, 3 × 10^−8^ and 7 × 10^−8^ M for **8**, **7**, and **6**, respectively. This was further improved by a factor of approximatively 10 by using Cremophor EL based emulsions.

Brasseur *et al*. [107] conjugated polyethylene glycol [PEG; molecular weight (M.W.) of 2,000] or polyvinylalcohol (PVA; 13,000–23,000) axially to AlPc (Figure 5). The resulting steric hindrance combined with the increased water-solubility provided by the axial polymeric ligands, was expected to enhance the efficacy of such AlPc derivatives. *In vitro*, after 1 h of incubation, the hydrophobic **6** was 3 times more potent in EMT-6 cells to induce 90% of cell death than its axially derivatized counterparts. Interestingly, in *in vivo* experiments, **10** showed the longest half-life (6.8 h *vs.* 23 min for AlPc-PEG and 2.6 h for **6** in Cremophor EL), the lowest retention in the liver, spleen and lung and most importantly the highest tumor accumulation. Tumor-to-skin and a tumor-to-muscle ratios of 12 and 27 respectively after 24 h post injection in EMT-6 tumor bearing mice have been reported whereas, **9** exhibited the lowest half-life and accumulation in tumor. Thus, the axial substitution of AlPc with PVA could exert the same efficiency as **6** formulated in Cremophor thereby avoiding potential risk factors such as anaphylactic reactions [108].

#### 2.1.7. AlClPc-Formulation

Besides the use of Cremophor EL based emulsions, some efforts have been reported on the formulation of AlClPc (**6**) in suitable delivery vehicles aiming at improving the photodynamic outcome of AlClPc-based PDT such as polymeric pH-sensitive micelles.

Micelles are usually aimed at entrapping hydrophobic drugs in their lipophilic core mostly to enhance their bioavailability and to make them suitable for systemic administration. Furthermore, owing the Enhanced Permeability and Retention effect (EPR effect), micelles from 5 to 50–100 nm allows the preferential accumulation of drug into tumors [109,110]. Indeed, abnormalities at tumor sites *i.e*., defective and leaky neovasculature, low lymphatic drainage and limited venous return, enable drug accumulation and retention in tumors [111,112]. Moreover, pH-sensitive micelles are expected to enable specific tumor delivery of the therapeutic agent based on the physiological differences between healthy and cancerous tissues [113].

It was demonstrated *in vitro* that pH-sensitive Polymeric Micelles (PM) of N-isopropylacrylamide (NIPAM) copolymers loaded with **6** were photodynamically more active than **6** in a Cremophor formulation on EMT-6 cells [114]. Furthermore, the terminally alkylated NIPAM copolymers loaded with **6** were less efficient *in vitro* than its randomly alkylated counterpart.

*In vivo*, **6** encapsulated in PM exhibited a rapid clearance and a low accumulation compared to the Cremophor formulation. However, despite these unfavorable pharmacokinetic characteristics, **6** in PM showed a “similar phototoxic activity to that of the Cremophor formulation” [115].

Trials to decrease PM clearance and thus improve their phototoxicity by incorporating hydrophilic *N*-vinyl-2-pyrrolidone (VP) units into NIPAM copolymers failed; mainly due to increased accumulation in the lungs and unchanged uptake by tumor cells [116].

The intrinsic insolubility of AlClPc in aqueous media represents certainly a drawback if systemic administration of the drug is desired. However, in case of topical application, for the treatment of non-melanoma skin cancer the aggregation of the Pc can be circumvented by the use of pharmaceutically acceptable solvents that both enable the dissolution of the drug and additionally enhance the permeability of the skin such as DMSO [117]. In a study conducted by Kyriazi *et al*. [118], AlClPc was diluted in a mixture of DMSO, Tween 80 and water and tested on albino mice (SKH-HR1) and SKH-HR2 (with melanin) bearing UV-induced skin carcinomas. Irradiation was performed both on the tumor and also on the normal skin surrounding the tumor. Optimal tumor response in terms of “highest percentage of mice in complete remission” was obtained, with a light dose of 150 J/cm^2^ and a fluence rate of 75 W/cm^2^. Furthermore, the formulation enabled a 40 times more selective uptake of the drug by the tumors compared to normal skin after 1 h post-administration. Interestingly, better therapeutic outcome was achieved when lower fluence rates were applied. This observation is in agreement with other studies conducted on mTHPC and 5-ALA photosensitisers and was related to the lower consumption of oxygen under these conditions [119,120].

### 2.2. Zinc Based Phthalocyanines

#### 2.2.1. ZnPc—SAR

**Figure 6:**
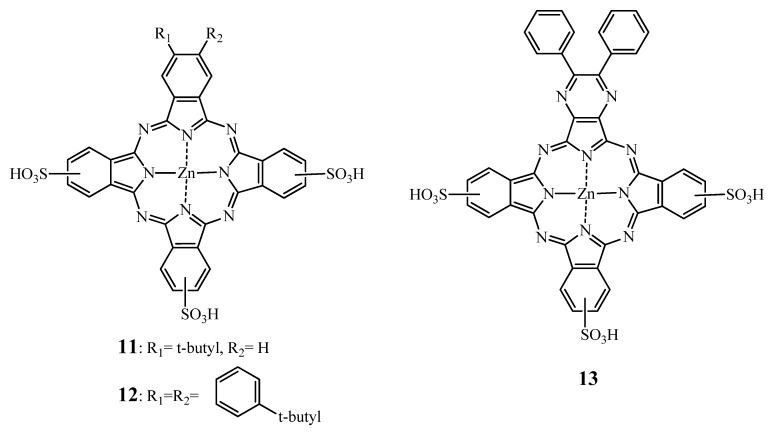
Structures of compounds **11**, **12** and **13** [122].

As mentionned in Section 2.1.1, providing permanent negative charges through sulfonation to Pcs increases their solubility in aqueous media. Wöhrle *et al*. reported [121] that amphiphilically sulfonated ZnPcs such as the mono- and disulfonated phthalocyanines are more potent and effective than their tri- and tetrasulfonated counterparts *in vivo*. Comparing Zn and Al substituted analogs, they found the following pattern of photodynamic efficacy: AlPcS_2_ ≈ ZnPcS_1_ > AlPcS_1_ > AlPcS_3_ ≈ ZnPcS_2_ > AlPcS_4_ > ZnPcS_4_. In 1997, Kudrevich *et al*. [122] conducted *in vitro* and *in vivo* experiments on Balb/c mice bearing EMT-6 tumors using a series of trisulfonated, amphiphilic water-soluble ZnPc derivatives (Figure 6).

*In vitro* compounds **11** and **12** were two and more than five times more potent than the tri and tetrasulfonated zinc phthalocyanines, respectively, whereas compound **13**, exhibited the lowest phototoxicity probably due to its poor cellular uptake caused by the “bulky diphenylpyrazino substituent”. *In vivo*, no tumor regression could be noticed with **13** and ZnPcS_3_. However, in almost 90% of the mice treated with 5 μmol/kg of compounds **11**, **12**, and ZnPcS_4_ no palpable tumors have been detected three weeks post-PDT. It was suggested that the amphiphilic character of **11** and **12** decreases the aggregation tendency, thus increasing photoactivity and tumor uptake. Moreover, due to the higher conjugation extent, a bathochromic shift of the Q-band (*i.e*., 706 nm) was induced.

Based on the experiments evaluating the impact of amphiphilicity on photodynamic activity with aluminium sulfonated phthalocyanines, there have been numerous attempts to increase the amphilic character of zinc sulfonated phthalocyanines. In one of their studies, Cauchon *et al*. [123] compared the photodynamic efficiency of different trisulfonated phthalocyanines bearing one alkyl chain containing between n = 2 and 16 carbon atoms (ZnPcS_3_(C_n_)).

*In vitro* studies revealed that ZnPcS_3_(C_9_) exhibited the highest cellular uptake and photocytotoxic effect on EMT-6 cells, while the other hydrocarbon substituted phthalocyanines were less efficient. The structure-activity relationship to induce 90% cell death of these compounds followed a typical hyperbolic curve also observed for other homologous series of photosensitizers [124]. However, the most potent compound in this study was the disulfonated zinc phthalocyanine with two sulfonic groups positioned adjacently despite a lower cellular uptake which can be explained by different intracellular localization upon internalization.

**Figure 7:**
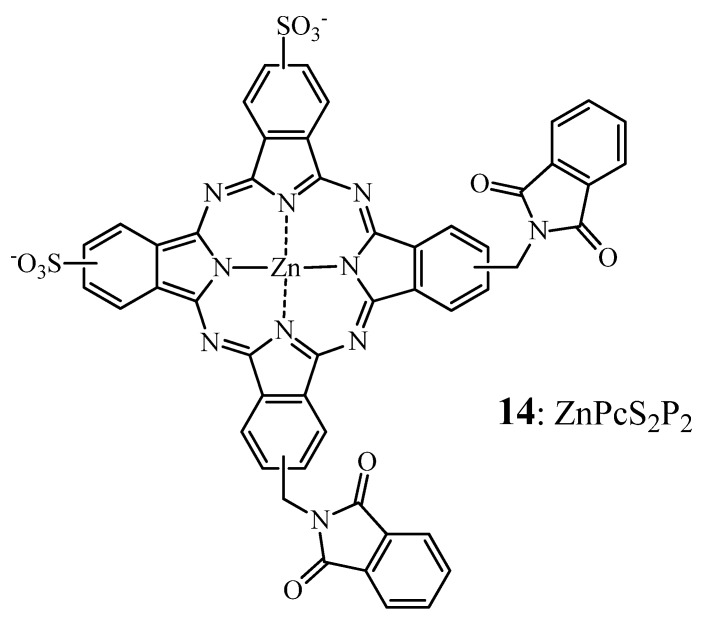
Chemical structure of phthalocyanine **14***i.e*., ZnPcS_2_P_2_.

In a series of different ZnPcs, disulfo-di-phthalimidomethyl zinc phthalocyanine (ZnPcS_2_P_2_ = **14**) (Figure 7) has been shown the highest photodynamic activity in Human myelogenous leukemia HL60 cells, K562, FGC85, SGC7901 and LCC carcinoma cell lines [125,126,127]. Moreover, when administered to mice bearing S180 and U14 solid tumors [126] in a Cremophor-based emulsion, the tumor weight in PDT treated animals decreased compared to the controls. The highest inhibitory rate was achieved with a drug dose of 2 mg/kg in both models. Moreover, no acute toxicity was reported after intravenous or intraperitoneal administration of **14** with drug dose up to 100 mg/kg [126]. The repeated-dose toxicity of this compound was assessed a few years later in healthy Wistar rats [128] using the same delivery system. Interestingly, an acute toxicity of **14** was found at a dose of approximatively 52 mg/kg based on their investigations on mice [126] and on an “anticipated human clinical application” (data not disclosed).

It was reported that a 10 times repeated administration of 4 mg/kg, which corresponds to 100 times that anticipated clinical “therapeutic” dose by the authors, induced notable hepatic and spleen damage.

Besides sulfonation, another means to enhance the solubility of Pcs in aqueous media and increase their amphiphilicity is hydroxylation. Boyle *et al*. [129] and Hu *et al*. [130], reported the synthesis and evaluation of several hydroxysubstituted zinc phthalocyanines.

**Figure 8:**
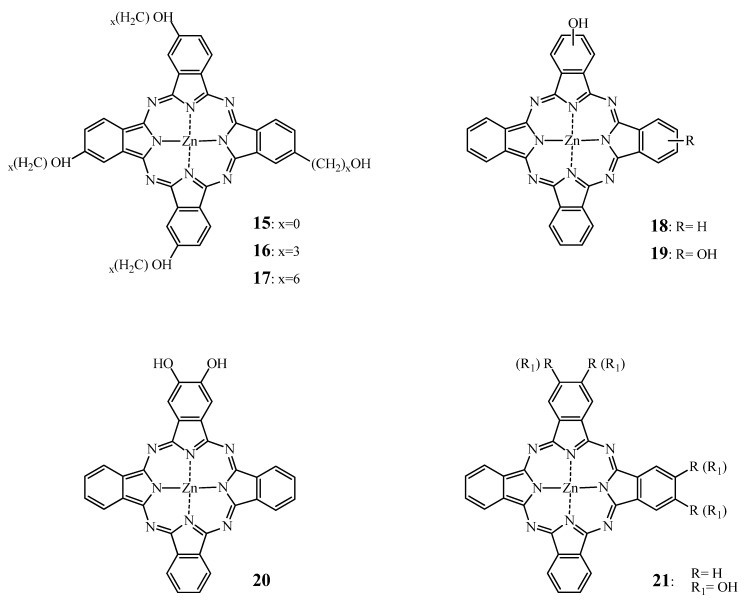
Chemical structure of hydroxylated phthalocyanines **15–21**.

When incorporated in Cremophor EL emulsions, all compounds showed strong photodynamic action both *in vitro* and *in vivo* except for the directly substituted tetrahydroxyphthalocyanine **15** (Figure 8). Indeed, Boyle *et al*. [129] showed that **15** was phototoxic *in vitro* on Chinese hamster lung fibroblasts V79; but failed *in vivo* on Balb/c mice bearing EMT-6 tumors, to induce any perceptible tumor response. Meanwhile, the tetra(3-hydroxypropyl) zinc phthalocyanine **16** (Figure 8) and tetra(6-hydroxyhexyl)zinc phthalocyanine **17** (Figure 8) induced tumor cure as measured by tumor necrosis within 48 h and no tumor regrowth up to 30 days at 0.5 μmol/kg and 1 μmol/kg (1.0 mg/kg), respectively, when applied intravenously. Compared to a mixture of mono, di, tri and tetrasulfonated ZnPcs, **16** and **17** were ten and five times more potent than ZnPcS. Furthermore, it seemed that the hydroxyderivatives function via vascular shutdown rather than direct cell killing as observed for the sulfonated zinc phthalocyanines.

Moreover, Winkelman *et al*. [131] postulated that a critical distance of 1.2 nm is required between oxygen atoms of sulfonate, carboxyl or hydroxyl substituents and the core of phthalocyanines or porphyrins in order to present biological activity. This hypothesis could potentially explain the low photoactivity exhibited by **15** as well as the phototoxicity of the alkylhydroxy ZnPc derivatives.

A few years later, Hu *et al*. [130] tested mono-, di- and tri-substituted hydroxy ZnPcs derivatives **18**–**21** (Figure 8) formulated in Cremophor emulsions in EMT-6 tumor cells *in vitro* and *in vivo*. It was observed that the phototoxicity decreased with the introduction of hydroxyl groups (*i.e*., **15** *vs.***20**) and that adjacent positioning of the hydroxyl functions such as in **21** increased Pcs phototoxic effect. 

**Figure 9:**
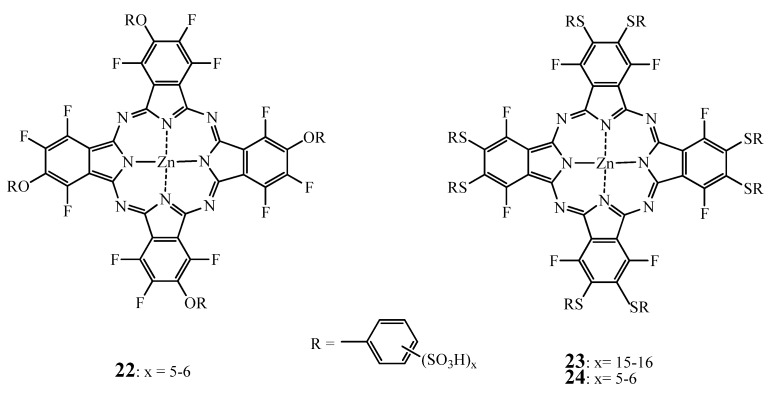
Structure of the fluorinated ZnPc **22**, **23** and **24**.

A third possible chemical modulation of ZnPc consists in fluorination of the PS. Several articles report the synthesis and evaluation of this class of ZnPcs [132,133] (Figure 9). The main reasons being that fluorination increases the water solubility and triplet state quantum yield of the phthalocyanine while maintaining similar behavior in biological medias as hydrogen atoms [134].

**Figure 10:**
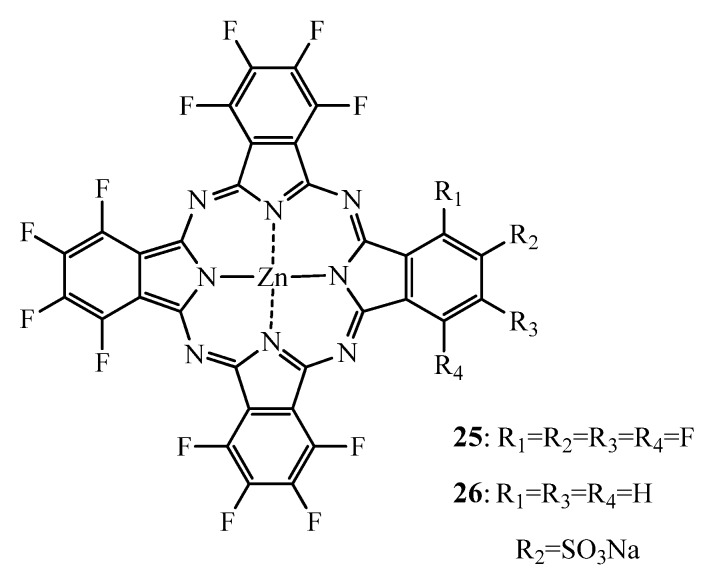
Structure of the zinc hexadecafluorinated Pc **25** and its monosulfonated analogue **26**.

The most extensive studies on fluorinated ZnPc in PDT have been performed by the van Lier group (using hexadecafluorinated zinc phthalocyanine (ZnPcF_16_)(**25**, Figure 10) [135,136,137,138].

In their most recent publication on this subject, Allémann *et al*. [137] compared Cremophor based emulsion of compounds **25** and **26**. It is noteworthy to mention that Pc **26**, bears one sulfonated group to provide some amphiphilicity, thus, probably improving cell penetration. To BALB/c mice bearing EMT-6 tumor allografts, 1 μmol/kg of **25** or **26** were applied and their bioavailability as well as their PDT effect has been assessed. After one week, the area under the plasma concentration as a function of time of **25** and **26** were 237 and 183 nmol∙h/g with half-lives of 9.25 and 12 h, respectively. The highest PS tumor accumulation was recorded 24 h post-injection for both Pcs. However, **26** exhibited better tumor selectivity with reported tumor-to-muscle and tumor-to-skin ratios of approximatively 13 and 4 for **26** and, 7 and 2.5 for **25**, respectively. Strikingly, a 66% tumor response was recorded for **26** at 1 μmol/kg for a light dose of 100 J/cm^2^ but was associated with 33% mortality. Hence, it was considered that the best PDT results (*i.e*., tumor response and animal viability) were obtained using 0.1 μmol/kg of **26** irradiated at 400 J/cm^2^. As compared to previous results reported by Boyle *et al*. [138], **26** is twenty times more phototoxic than **25** while displaying equivalent tumor uptake. The impressive **26** phototoxicity observed was attributed to “extensive cellular damage”. In tumor free rats, **26** formulated in Cremophor was devoid of any lethality after irradiation. Alterations of tumor surrounding tissues and extensive edema were reported, but were associated with rapid recovery [137]. A proposed way to avoid these collateral damages is to tune its biodistribution and elimination using different formulations forms, such as PEG-coated nanoparticles (NP). In Section 2.2.3 of this review, formulation of **25** in NP is further discussed.

Allémann *et al*. [137] also investigated the influence of incubation media on PDT efficiency of ZnPc *in vitro* by comparing 1-methyl-2-pyrrolidinone to pyridine. Dilution of Cremophor emulsion of ZnPc at 10 μM in 1-methyl-2-pyrrolidinone solution led to complete loss of *in vitro* PDT efficacy presumably due precipitation and aggregation as noticed by an altered absorption spectrum. This observation confirmed earlier *in vivo* studies by Boyle *et al*. [138] that obtained no significant tumor response in BALB/c mice bearing EMT-6 tumors when ZnPc was diluted in 1-methyl-2-pyrrolidinone. In the meantime, complete tumor ablation was achieved when pyridine was used to dilute ZnPc Cremophor-based emulsion at a drug dose of 2 μmol/kg. The authors assumed a possible “coordination of pyridine to the axial ligands of the central metal ion” resulting in an increase photodynamic activity.

Choi *et al*. and Liu *et al*. [139,140] reported recent *in vitro* investigation*s* on the influence of glycosylated ZnPc based on the strategy to concomitantly increase the water-solubility and the selectivity of these compounds through targeting of glucose transporter [141,142]. In accordance with zinc sulfonated phthalocyanines, tetra-glycosylated ZnPc derivatives were substantially less photoactive than the mono-glycosylated and di-glucosylated ZnPcs derivatives *in vitro* [139,140]. Moreover, the α and β positioning of the glycose substituents seemed to influence the tendency to aggregate and consequently affects their phototoxicity. Indeed, α substitution appeared to prevent Pc aggregation as compared to the β positioned analogues. Promising phototoxicities in the nanomolar range were achieved in HT29 and HepG2 cells glucosylated di- α substituted ZnPc.

**Figure 11:**
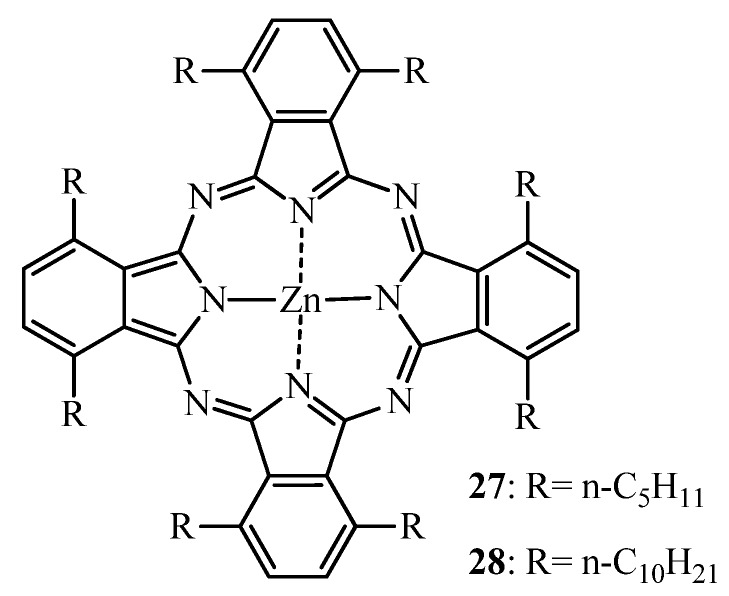
Chemical structure of octapentyl and octadecyl ZnPcs **27** and **28**.

Ometto *et al*. [143] as well as Fabris *et al*. [144] have tested octapentyl (**27**) and octadecyl (**28**) substituted ZnPc (Figure 11). Administered intramuscularly to MS-2 fibrosarcoma bearing Balb/c mice in a Cremophor emulsion both compounds were highly selective for the tumor tissue. Limited skin photosensitization was confirmed with healthy Balb/c mice under the same experimental conditions. The strong binding of both compounds to LDL is presumably responsible for this selectivity [145]. Both **27** and **28** induced a shrinkage of the tumor volume after PDT [143,144]. However, both derivatives accumulated to a higher extend in the liver and spleen than in the tumor even one week post-PDT, presumably because the bile-gut is the primary elimination pathway characteristic for lipidic drug delivery systems [143,146]. 

**Figure 12:**
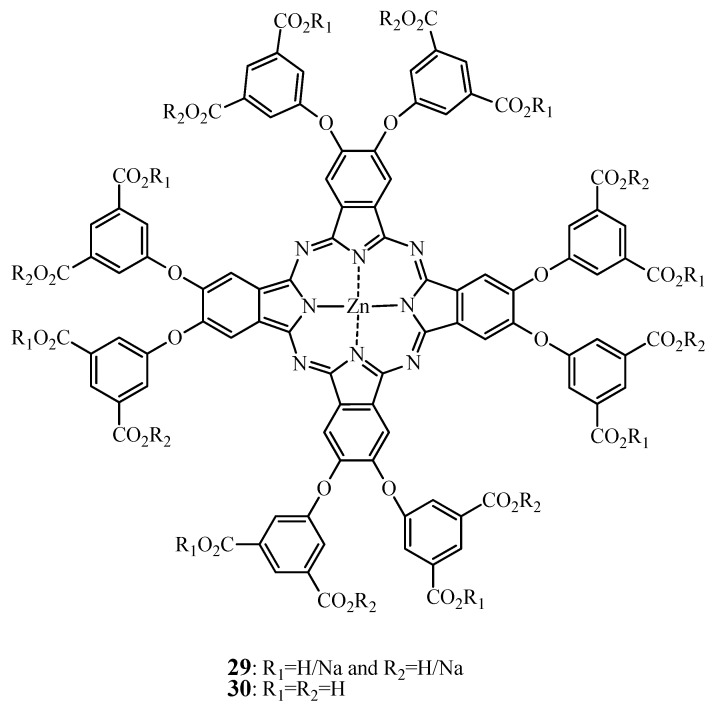
Chemical structure of phthalocyanines 29 and 30.

Liu *et al*. have achieved synthesis and *in vitro* evaluation of zinc octa[(biscarboxylate)-phenoxy]phthalocyanine and its sodium salts [147] (*i.e*, compounds **29** and **30**, see Figure 12).

This structural extension with phenylcarboxylate groups resulted in a diminished stacking and aggregation tendency, due to possible non-planar orientation of this group as well as extended “inter-ring distance”. However, at physiological pH, only **29** was water-soluble. The authors presented this aspect as the major drawback of **29** to HEp2 cell penetration, whereas the more hydrophobic **30** was taken up to a higher extent. However, cell penetration could have been also hampered by the presence of negative charges on **29** at physiological pH. Despite differences in cellular uptake both Pcs displayed similar but moderate phototoxicities. However, this low potential for photosensitization of biological materials can be efficiently used for fluorescence diagnostics. Very recently ZnPc similar to **29** zinc tetra[(monocarboxylate)phenoxy]phthalocyanine has been exploited for the use in PCR analysis [64]. This compound had similar but narrower absorption and emission bands in the NIR as compared to a conventional cyanine dye. Most importantly, the Pcs dyes were thermally and chemically stable and showed essentially no photobleaching.

The outstanding tendency of Pcs to form photoinactive aggregates can also be exploited for the design of “molecular beacons”. These compounds are powerful tools for real time detection of RNA/DNA *in vitro* and *in vivo*. In molecular beacons, a quencher/donor pair is positioned on the distal ends of a short, hairpin forming oligonucleotide. In absence of a complementary sequence the close proximity of the fluorophore and its corresponding quencher makes the molecular beacon optically silent. However, hybridization with the complementary sequence then restores the fluorescence of the reporter. Despite their high specificity the sensitivity of conventional molecular beacons is often compromised by a poor sensitivity provided by insufficient quenching. Futhermore, longer observation periods are often impeded by most fluorescent dyes’ strong photobleaching. Therefore, in an earlier study the same group reported the use of a pair of Pc similar in structure to ZnPc in molecular beacons [148,149]. After optimization of the reaction conditions good yields for molecular beacons were achieved. However, since Pcs have long interaction ranges the authors had to use longer oligonucleotide sequences as complementary sequence to observe a fluorescence increase. A signal to background ration as high as 59 was reported. Furthermore, the perfect matched complementary sequence was five times more fluorescent than a single base mismatch. Therefore, water soluble Pcs can be efficiently employed as fluorescence reporters *in vitro* but their whole potential in this area remains to be demonstrated.

**Figure 13:**
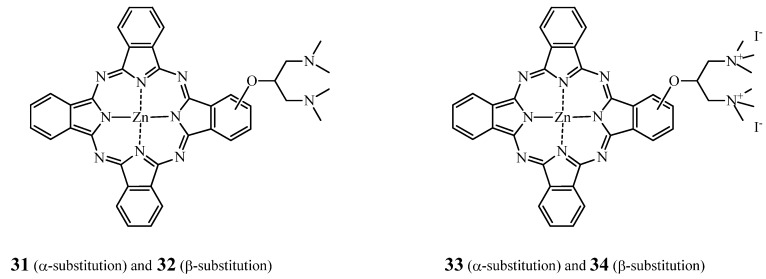
Structure of compounds **31–34**.

Lo and co-workers reported the photoactivity of monosubstituted ZnPc with a 1,3-bis(dimethyl-amino)-2-propoxy group at the α or β position (Figure 13), “and the corresponding di-*N*-methylated derivatives” on human colorectal carcinoma cells HT29 [150]. The α and β positioning is an analogy to the α and β positions for sugar moieties. Indeed in sugars, α and β correspond to lower and upper position respectively of the hydroxylic group on the anomeric carbon (C1) of the cyclic sugar moiety, the cylcle defining the referential plane [151].

The absorption and fluorescence spectra showed a lower aggregation tendency for the β-substituted compounds as compared to their α-substituted analogues. Consequently, the IC_50_ of **32** and **34** were 0.15 and 0.08 μM, respectively, while **31** and **33** induced 50% of cell inactivation at doses of 0.48 and 0.64 μM, respectively. 

Like AlPcs, PEGylated ZnPcs have also been designed aiming at increasing their water solubility [152]. Liu *et al*. have prepared a series of asymmetrically substituted ZnPcs using methylated polyethylene glycol. However, these compounds were essentially not water soluble. Therefore, just recently the synthesis of tetra and octa substituted PEGylated ZnPcs has been reported [153]. In these compounds the terminal methyl group of the tetraethylene glycol side chains has been omitted in order to provide a higher hydrophilicity through hydroxyl end groups. Despite these modifications, aggregation in water was still observed that could be circumvented by the addition of Triton-X100. The less aggregated compound that was non-peripherally substituted with polyethylene glycol showed a strong bathochromic shift of more than 20 nm. But *in vitro* evaluation showed an IC_50_ value that was in the order of two orders of magnitude higher than reported for the methylated counterparts. Recently, Ng and co-workers reported the synthesis and efficient photocytotoxic effect of several pegylated ZnPcs where IC_50_ values ranged from 0.25 to 3.72 μM on HT29 and HepG2 cells [154].

**Figure 14:**
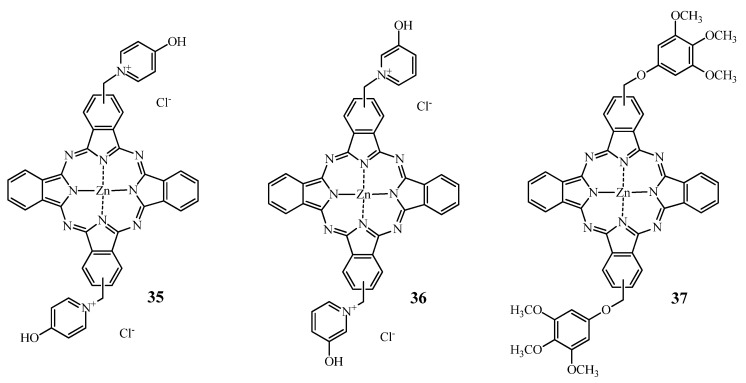
Structure of compounds **35**, **36** and **37**.

The net charge of pharmacological compounds is of importance with respect to the pharmacokinetic properties of a drug as well as in terms of uptake and intracellular localization. Several studies report the impact of the charge on Pc-mediated PDT. Banfi *et al*. [155] described the synthesis and *in vitro* assessment of some ZnPc derivatives on human colon adenocarcinoma cells HCT116. The most potent ZnPc derivatives among the synthesized series are shown in Figure 14.

Compound **35** was approximately five times more potent than compound **36** and approximately eight times more efficient than compound **37**.

**Figure 15:**
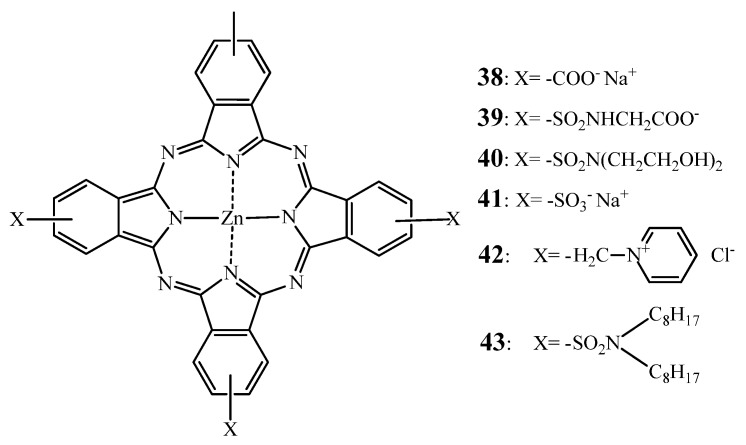
Chemical structure of phthalocyanines **38–43**.

**Figure 16:**
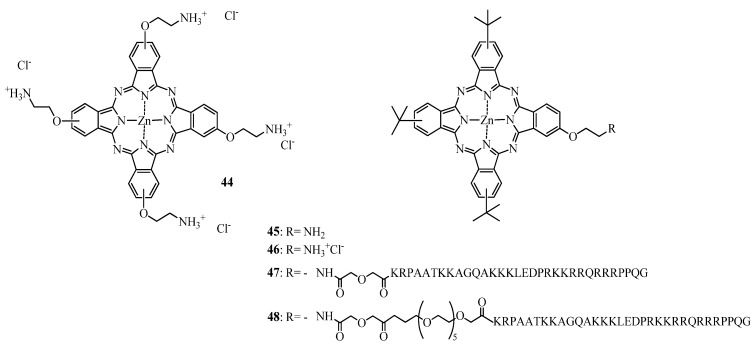
Chemical structure of compounds **44–48**.

Brown and coworkers [156,157] reported that the cationic pyridinium ZnPc **42** (see Figure 15) was more phototoxic to RIF-1 cells than the anionic tetraglycine analogue **39** or the neutral hydrophobic tetradioctylamine **43** [156]. *In vivo* all the tested compounds induced partial tumor regression, but only compound **42** led to complete remission, most presumably through vascular occlusion in the irradiated areas. This observation is in agreement with a study of Sibrian-Vazques *et al*. that showed that the cellular uptake of **44** was higher and faster than **46** (see Figure 16) [158].

#### 2.2.2. Tumor Targeting with Zinc Based Pc-Conjugates

In the same report the preparation and *in vitro* evaluation of zinc based phthalocyanines conjugates with cell-penetrating peptides (CPP) (see Figure 16) was described.

Conjugates **47** and **48** were taken up by HEp2 cells to a higher extent than the unconjugated ZnPc. The introduction of short polyethylene glycol linker between the ZnPc and CPP has been shown to further increase the uptake. At a light dose of approximately 1 J/cm^2^, **47** and **48** were the most active compounds, with IC_50_ values of 1.44 μM, 1.87 μM, respectively. In contrast, **44** did not alter the cell viability at doses up to 10 μM. 

#### 2.2.3. Formulation of ZnPcs

Phototoxicity and fluorescence of Pcs is mostly hampered by their low water-solubility and tendency to aggregate. Their incorporation into suitable and biodegrable formulations is commonly accepted as a necessity in the development of new therapeutics. Indeed, development of drug delivery systems such as liposomes, micelles and nanoparticles could improve the unfavorable biodistribution of free Pcs (*i.e*., improvement of PS pharmacokinetic properties, better targeting of diseased tissues due to size of the particles, association to serum proteins and specific activation of the PS through localized delivery of the PS) as well as avoidance of aggregation and loss of phototoxic activity/fluorescence which should result in a better therapeutic outcome (see following paragraphs). 

Zinc based phthalocyanines have been mostly evaluated as liposomal formulations. Liposomes are phospholipids vesicles that present the advantage to entrap either hydrophilic drugs in the core of the phospholipid vesicle or hydrophobic drugs within the lipid membrane. Incorporation of drugs into liposomes results in passive targeting of tumors [159,160]. In studies reported in this review, only small unilamellar vesicles (*i.e*., one phospholipid bilayer) were used.

Liposomal formulation of ZnPc showed photocytotoxic effect *in vitro* on a large variety of tumor cell lines [161,162,163]. The drug CGP55847 commercialized by CIBA has been the first Pc reaching clinical trials although no clinical results have been reported. Ben-Hur and Chan reported that the “early phase I/II clinical studies […] were discontinued but not for medical reasons” [164].

*In vivo*, ZnPc being incorporated in dipalmitoyl-phosphatidylcholine (DPPC) liposomes showed complete response at drug doses as low as 0.14 mg/kg in mice inoculated with MS-2 fibrosarcoma. At a slightly lower dose of 0.12 mg/kg (i.v.), ZnPc was found to be “associated exclusively with lipoprotein fraction”, *i.e*., whether it was complexed with low density lipoproteins (LDL) or incorporated into liposomal structures, and 70% of the drug was cleared from the body within 12 h independently from its carrier system, while the remaining ZnPc was slowly eliminated. However, ZnPc associated with LDL presented a higher selectivity toward tumor than ZnPc liposomes with a tumor-to-liver ratio of 2.27 and 1.04 and a tumor-to-muscle ratio of 4.20 and 3.84 after 24 h post-injection, respectively. Nonetheless, the selectivity was somewhat limited by the redistribution of the photosensitizer among other lipoproteins, especially high density lipoproteins. As liposomal formulations of benzoporphyrin derivative, liposomal CGP55847 seems to be transferred to serum lipoproteins and more predominantly to low density lipoproteins [165,166,167,168,169] explaining the propensity of the PS to selectively accumulate in tumors [163,170]. Rodal *et al*. suggested that the observed internalization of ZnPc was not following the endocytotic pathway of LDL but rather occurred via diffusion through the cellular membrane after binding to the LDL receptor [161].

*In vivo* studies on different liposomal formulations consisting of a mixture 1-palmitoyl-2-oleoyl-sn-glycero-3-phosphocholine (POPC) and 1,2-dioleoyl-sn-glycero-3-phospho-L-serine (OOPS) revealed that intratumoral distribution pattern of ZnPc in C57BL/6 mice bearing Ehrlich carcinomas or B16 melanomas was a time dependent process [171]. In agreement with van Leengoed *et al*. [172], 3 h after intravenous injection of 0.5 mg/kg of ZnPc, the photosensitizer was present in and around the tumor vasculature but not 24 h post-injection. Indeed, using a dorsal skinfold chamber model, van Leengoed *et al*. [172], could detect vascular occlusion within five minutes after photoactivation which resolved 30 min after irradiation. Moreover, maximal vasculature damage and tumor-to-muscle ratio from 8:1 to 14:1 have been reported for this liposomal delivery system [173].

Nanoparticles are another type of drug delivery system that enables the incorporation of higher drug doses of hydrophobic drugs. Passive targeting of tumors is also achieved due to their size (*i.e*., below 1,000 nm, generally 200 nm) through EPR effect. A strategy to increase the circulation time of the nanoparticles as well as other drug delivery systems is the incorporation of PEG moieties on their surface. It is commonly accepted that PEGylation creates hydrophilic barrier that reduces the immunogenicity of the drug delivery system and lowers its clearance from the body [174,175,176].

Polylactic acid (PLA) based nanoparticular formulations of ZnPc were investigated by Allémann *et al*. [135,136]. The coating of nanoparticles with PEG moieties resulted in the decrease of the reticuloendothelial system and an increase in the PS tumor retention [135]. Incorporation of **25** (Figure 10) into PEG coated nanoparticles or using a Cremophor EL based emulsion increased the bioavailability as compared to uncoated-NP by a factor of four in BALB/c mice bearing EMT-6 tumors. The tumor-to-skin and tumor-to-muscle ratios for the PEG-NP and Cremophor based formulation were 2 to 21, respectively, with a maximum concentration in tumor 48 h post-injection [135]. However, the nanoparticular drug delivery system was nearly five times more efficient for PDT-mediated treatment of mice bearing EMT-6 tumors [136]. This is in agreement with a study reported by Fadel *et al*. [177] describing that tumor bearing animals treated with ZnPcs laden poly(lactic-coglycolic acid) nanoparticles showed the best PDT outcome, highest tumor growth delay and longest survival times as compared to mice treated with the free PS. 

An interesting approach was recently described by Kataoka and co-workers [178,179,180,181] where dendrimer based zinc phthalocyanines showed promising results in PDT and PCI applications. Indeed, they could manage to prepare anionic dendrimer zinc phthalocyanine (DPc) incorporated into positively charged poly(ethylene glycol)-poly(L-lysine) (PEG-PLL) block copolymeric micelles referred to as polyion complex micelles (PIC) [180]. The substitution of large dendritic parts avoids the aggregation and increases their water solubility [181]. These micelles were tested both *in vitro* on human lung adenocarcinoma cells A549 and *in vivo* on (Balb/c nu/nu) mice bearing A549 tumors. It was shown that 7.6 times more of encapsulated DPc in micelles (DPc/m) was taken up and that these are 78 times more phototoxic *in vitro* than free DPc. In addition, subsequent *in vivo* studies confirmed the benefits of micelle encapsulation of DPc by delayed tumor growth and limited skin photosensitization.

Another interesting approach for drug delivery of hydrophobic PS is their conjugation to cyclodextrins. This strategy enables the water-solubilisation of lipophilic PS and thus its systemic administration at high concentrations. In one report, Baugh and al. proceeded to the conjugation of zinc based phthalocyanines to cyclodextrins. The double carbon bounding linkage between the dimers was cleaved, upon light activation and in presence of oxygen; resulting in the release of the Pc. Hence, this approach could be a promising systemic carrier of hydrophobic PS for photodynamic therapy [182,183].

Formulation of ZnPcs in gels or (micro)emulsions could be of interest in topical application. Indeed, several studies reported improved photophysical and aggregation properties of ZnPcs [184,185] as well as successful delivery to the skin of sulfonated ZnPc either via simple skin penetration [184] or through iontophoresis [186].

### 2.3. Silicon Based Phthalocyanines: Pc 4 and its Analogues

#### 2.3.1. SiPc-SAR

Several silicon based phthalocyanines (SiPc) mostly aiming at revealing the influence of axial ligands have been proposed and tested both *in vitro* and *in vivo*. Probably, the most famous representative of these compounds with some commercial value is “La Jolla Blue”. Water-solubility of this dye absorbing at 680 nm as well as prevention of aggregation is provided by two axial polyethylene oxide moieties. Then, two peripherial carboxy groups at the macrocycle can be used for the coupling to biological molecules such as antibodies. This has been used for the design of an antibody for an FDA cleared *in vitro* immunofluorescence assay. 

**Figure 17:**
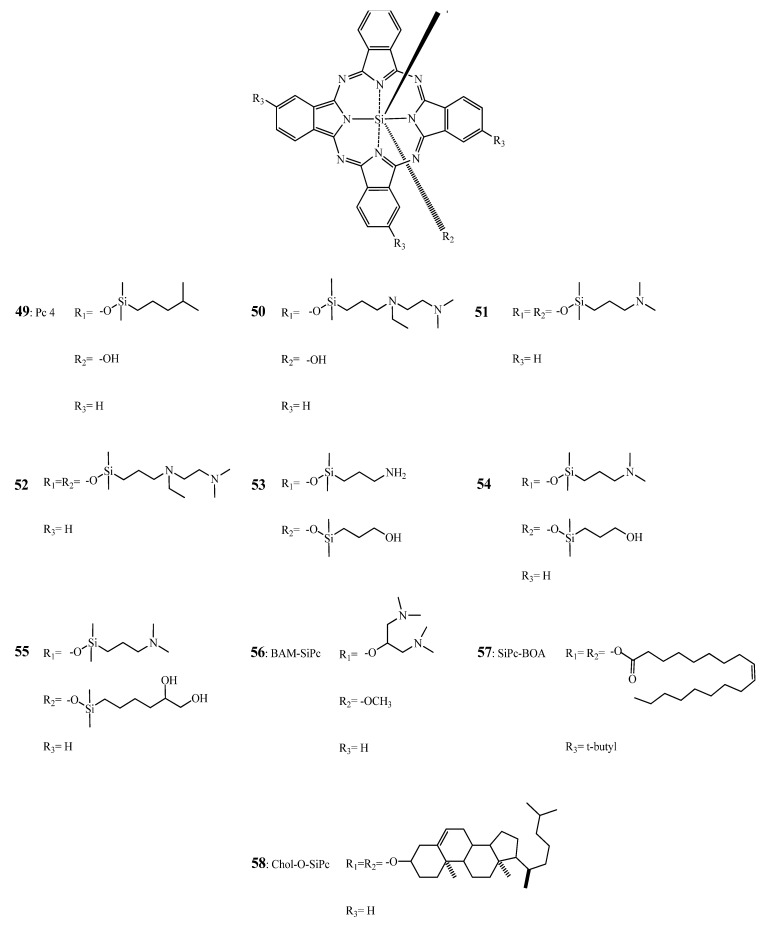
Chemical structure of Pc 4 (**49**) and derivatives **50–58** [187,188,189,190,191,192,193,194].

It has been shown by He *et al*. [187], that silicon based phthalocyanines with short aminosiloxy ligands [49 (Pc 4) and 51] are more phototoxic effect in Chinese hamster lung fibroblasts V79 and Murine leukemic lymphoblasts L5178Y-R as compared to compounds such as **50** and **52** with longer axial ligands (see Figure 17).

In another report [194], the PDT effects of these compounds *in vivo* on C3H/HeN mice bearing RIF-1 tumors were examined. The phthalocyanines were solubilized in a Cremophor EL emulsion and light was given 24 h post Pc injection. Except **52**, all axially substituted Si-Pc resulted in a complete remission. The authors suggested that **51**-mediated PDT may induce cellular destruction via a slower mechanism than Pc 4-mediated PDT.

In 2009, Rodriguez *et al*. [188], compared Pc 4 to compounds **51**, **53**, **54** and **55** on the human breast cancer cell line MCF-7c3. These analogues showed, among other features, higher cellular uptake and phototoxicity than Pc 4. Biaxial substitution reduced aggregation which, in turn, can explain their higher photodynamic efficiency. Furthermore, the presence of a hydroxyl group at one side seems to enhance the phototoxicity of the SiPcs. However, as shown by confocal microscopy, except for **51**, Pc 4 analogues seem to act through a different destructive pathway due to their principle association with lysosomes rather than with mitochondria/endoplasmic reticulum. Interestingly, when clonogenic assays were performed **51** and Pc 4 had a similar behavior. In contrast, **51** exhibited a 4-fold lower IC_50_ in comparison to Pc 4 (**49**) as demonstrated by means of a MTT test. Using bi-axially substituted polyamine SiPcs with fluorescence quantum yields in aqueous media of 0.12–0.21, IC_50 _ranging from 450 to 1 nM have been reported in HT29 cells by Ng and co-workers [195].

Moreover, the same group of research developed several SiPcs substituted axially with β-cyclodextrins [196,197,198]. They could reach a photocytotoxic effect on human colon adenocarcinoma and hepatocarcinoma cells (HT29 and HepG2 respectively) with reported IC_50_ ranging from 21 nM to 1.32 μM. In addition, early *in vivo* studies conducted by Lau *et al*. [197] show promising results as drug doses as low as 1 μmol/kg for a light dose of 30 J/cm^2^ are efficient enough to suppress tumor growth in mice bearin HT29 xenografts.

Another promising approach used by the same group is the glucoconjugation of SiPcs [190,199]. In their report, Chan *et al*. [199] reported IC_50_ values as low as 6 nM on the same cell lines and could “retard tumor growth” in the same animal model.

Pc 4 has been developed at Case Western Reserve University at the beginning of the 90s. Since then Pc 4-mediated PDT has been reported effective *in vitro* against various tumor cell lines of different origin [187,200,201,202,203,204,205]. In experimental animal models for ovarian and colon cancer, no photodynamic effect was observed with doses of 0.4 mg/kg [202], whereas with doses of 0.6 or 1 mg/kg complete remission or at least significant tumor volume reduction occurred between 3 to 7 days post-PDT. Furthermore, Pc 4-PDT was associated with a delay of tumor regrowth from 9 [203] up to 90 days [202].

A recent study conducted on immunideficient mice bearing papillomas [206] induced by the administration of cottontail rabbit papillomavirus led to the conclusion that Pc 4 at a dose of 1 mg/kg and at a light dose of 150 J/cm^2^ given 48 h administration resulted in complete remission in 87% of the cases with no observable re-growth during 79 days. In addition, it was suggested that the rapid destruction observed in Pc 4-mediated PDT could be explained by the mitochondrial/endoplasmic reticulum [188] localization of Pc 4 mainly leading to apoptosis and necrosis [207,208,209]. However in some cases vascular occlusion was also reported [188].

Moreover, Anderson *et al*. [210] demonstrated that Pc 4 exhibited less skin photosensititization as compared to Photofrin®. Topically applied, Oleinick and co-workers established that Pc 4 is “effectively delivered into the human skin” [211] and was consequently investigated clinically. In these trials, Pc 4-mediated PDT was reported to be well tolerated in patients and could have promising application in mycosis fungoides treatment [212].

Recently, Fong and coworkers reported the synthesis and characterization of several silicon-based phthalocyanines [189,213,214,215,216,217]. Out of this series, BAM-SiPc **56** (see Figure 17) having an IC_50_ as low as 0.015 μM on HepG2, Hep3B, HT29 and J744 was the most potent. However, this compound was phototoxic on normal liver cells (*i.e*., WRL-68 with IC_50 _of 0.035 microM under the same experimental conditions) [189,192,218]. In nude mice bearing hepatocarcinoma HepG2 and colorectal adenocarcinoma HT29 tumors the same compound showed remission/regression and tumor growth delay 15 days after PDT at a drug dose of 1 μmol/kg [191].

Barge *et al*. [193] could also covalently link axial cholesterol moiety (Chol-O-SiPc) (Figure 17) and reported a seven fold higher potency of this derivative as compared to AlClPc (with IC_50_ of approximatively 8 nM) *in vitro*, on the pigmented melanoma cell lines M3Dau and SK-MEL-2.

#### 2.3.2. SiPc Formulations

Pc 4 is the most extensively studied silicon-based phthalocyanine. Due to its insolubility in physiological media, Pc 4 tends to aggregate with subsequent loss of photodynamic activity and altered biodistribution. In order to avoid these issues, Pc 4 was incorporated in oil-based formulations, PEG-PCL (polyethylene glycol-block-poly-ε-caprolactone), micelles, and nanoparticles.

In a recent study Master *et al*. [219] were able to encapsulate with 70% efficiency Pc 4 in PEG-PCL micelles. The authors observed that in human breast cancer cells (MCF-7c3), Pc 4 delivered in PEG-PCL micelles seemed to colocalize with lysosomes and mitochondria, while Pc 4 in solution accumulated mainly in mitochondria and endoplasmic reticulum as well as in Golgi apparatus. However, no different photocytotoxic effect was observed for the two formulations.

Pc 4 was also encapsulated into silica and gold nanoparticles known for their biocompatibility and their stability [220]. Zhao *et al*. [221] showed that encapsulation into silica nanoparticles of 25–30 nm diameter lead to enhanced photodynamic activity *in vitro* as compared to the free Pc 4. Moreover, Pc 4 silica nanoparticles were found to be less sensitive to photobleaching. The reported IC_50 _values for the nanoparticluar delivery system were around 5 nM for A375 cells and 10 nM for B16F10.

An *in vivo* biodistribution study with Pc 4 adsorbed on PEG coated gold nanoparticles revealed that Pc 4 gold nanoparticles accumulated in the tumor faster than Pc 4 formulated in Cremophor EL. While the peak concentration in the tumors was only reached after 2 days using Cremophor the gold nanoparticles containing Pc 4 reached this peak within 2 h. It was also observed that the singlet oxygen yield of the two formulations was identical suggesting that the nanoparticles protect Pc 4 until its release in the target tissue [222].

Li *et al*. reported the synthesis and PDT effect of a neutral and lipophilic tetra-*t*-butyl silica phthalocyanine bisoleate referred to as SiPc-BOA (Figure 17) [223]. It was noticed that axial substitution of the oleate moieties decreased the propensity of the photosensistizer to aggregate and enabled its binding to the lipidic layer (*i.e*., LDL delivery system). Using this strategy, Li *et al*. increased the loading of the LDL nanovesicules with a molar ratio of 400 SiPc-BOA per one LDL [223]. These SiPcBOA laden nanoparticles were 10 times more effective than the free compound in HepG2 cells suggesting a LDL receptor-mediated photodynamic effect. 

A multifunctional approach was proposed by Zheng *et al*. [224] through additional coupling of folic acid to lysine residues of the apolipoprotein B of the LDL part in SiPc-BOA-LDL conjugates. The validity of their hypothesis was tested in a system consisting of cells expressing the folate receptor (κB cells), those expressing the LDL receptor (HepG_2_ cells), and cells that lack the folate receptor (HT1080, CHO cells). An interesting review of the aforementioned results could be found in the following book [225].

## 3. Applications of Pcs in Fluorescence Imaging

Most investigations on phthalocyanine dyes in biomedicine focus on their application in PDT for cancer. However, just recently research was also extended to parasite and bacterial disease treatment such as leishmaniosis [226,227,228,229,230]. Furthermore, in this context, an at least as important research area, *i.e*., fluorescence imaging, is sometimes ignored. Fluorescence imaging allows the non-invasive detection of superficial disease in a preclinical or clinical setting. It is sensitive, can be perfomed in real time, and its resolution can be tuned to a molecular level. As few as a thousand cells can be detected by this methodology. Today, fluorescence imaging is successfully implemented in preclinical *in vitro* and *in vivo* research to specifically monitor the therapeutic outcome of new drugs in animals or to reveal disease mechanisms. Furthermore, it is used clinically for the detection of several diseases, including age-related macular degeneration [231] and cancer [232]. It has been shown to improve the detection rate of barely visible lesions, surgery and recurrence rate in malignant glioblastoma [233,234] and bladder cancer [235]. Despite the recent hype of fluorescent proteins introduced into molecular biology, exogenous fluorescent dyes [236,237] or exogenously-induced fluorescent dyes [238] still play an important role in this research area.

For optimal monitoring of diseases *in vivo* it will be advantageous that fluorescent dyes absorb and emit in the NIR region of the visble light spectrum in order to optimally penetrate into the tissue and induce only minimal autofluorescence upon excitation with light. Therefore, scientists have developed panoply of different NIR fluorescent dyes for the labeling of proteins, small bioactive peptides, or oligonucleotides [239]. Although there are many classes of NIR dyes including oxazines and rhodamines commercially available, the most commonly used belong to the class of cyanine dyes. One of these, indocyanine green, is approved for the detection of occult choroidal neovasculariuation secondary to age-related macular degeneration. Today these dyes cover an absorption spectrum ranging between 650 and 800 nm. As described above, phthalocyanine dyes can be fine-tuned to the desired absorption/emission wavelengths and can be made water-soluble [240]. Furthermore, they are chemically stable in most solvents and are not as prone to degradation in highly acidic/basic media. Some of these compounds can have a fluorescent quantum yield as high as 70% and are extremely photostable. And finally, compared to conventional cyanine dyes they have a long fluorescence lifetime and an extremely high extinction coefficient. Despite, these advantages there are only two compounds, La Jolla Blue^®^ and IRDye 700DX^®^, belonging to this interesting class commercially available. Although these compounds have relatively high fluorescence quatum yields, they can still be used as efficient photosensitizers, as demonstrated by, Mitsunaga *et al*. [58] by conjugation of IRDye 700DX^®^ to monoclonal antibodies targeting epidermal growth factor receptors. Using these conjugates efficient killing of tumor cells was demonstrated *in vitro* and *in vivo* when NIR was used as light source.

Their tendency to aggregate seems to be ideally suited for a relatively new class of fluorescent reporters referred to as “smart probes” [241]. This approach has been pioneered by Ralph Weissleder’s research group and is based on the selfquenching/dequenching paradigm [242]. In these compounds, typically several fluorescent dyes are coupled either directly or via chemically or enzymatically labile linker to a polymeric carrier. In their native state these reporters are optically silent due selfquenching of the fluorescent dyes. However, as soon as a chemical or enzymatic trigger results in the release of the fluorescent dye from its polymeric carrier this selfquenching is abrogated. Today, this methodology has been extensively used for the detection of proteolytic activity *in vivo*. One can deduce from the discussions on the photodynamic activity of aggregated Pc above, that these compounds can also be used in “smart probes” with a better selfquenching allowing the use of low molecular weight carriers with improved pharmacokinetics. Another phenomenon of aggregated Pc, will further favor their use in “smart probes”. As the extinction coefficient of the Q-band undergoes significant reduction in the aggregated form, these fluorescence reporters not only show reduced fluorescence intensities in the non-activated form but also are less sensitive to excitation. Furthermore, due to their lipophilicity, once cleaved, Pc dyes will stay longer at the activation site due to reduced clearance. Currently, we are evaluating this approach for simultaneously treating and monitoring diseases with Pc-based polymeric photosensitizer prodrugs [243,244,245].

## 4. Conclusions

Throughout this review, it has been shown that phthalocyanines are promising photosensitizers for photodynamic therapy applications. Some of them are even currently used in PDT (e.g., Photosens^®^) or tested in clinical trials (e.g., Pc 4). At a chemical level, it is concluded that Pcs’ mainly amphiphilic character leads to a higher efficiency *in vivo*. In addition, hampering their stacking via axial ligation and including positive charge(s) seems to influence their photocytotoxicity, by increasing their cellular uptake and internalization. It is also of major importance to consider their subsequent cellular (re)localization in order to understand and evaluate their photodynamic activity. Hence, by only considering their chemistry, phthalocyanines exhibit this flexibility, enabling further screening and investigations, which rises hope for cancer treatment. Perspectives such as chemical coupling of two photosensitizers as well as screening of new phthalocyanines are currently examined and could be interesting prospects for PDT cancer treatment [246,247,248,249].

From a pharmaceutical point of view, suitable and optimal formulations of Pcs can increase dramatically the therapeutic efficacy. Suitable drug delivery systems can act as a solubilizing matrix for the PS as well as a shield and protection from degradation.
